# Genome-wide identification and expression analysis of *SWEET* gene family in *Litchi chinensis* reveal the involvement of *LcSWEET2a/3b* in early seed development

**DOI:** 10.1186/s12870-019-2120-4

**Published:** 2019-11-14

**Authors:** Hanhan Xie, Dan Wang, Yaqi Qin, Anna Ma, Jiaxin Fu, Yonghua Qin, Guibing Hu, Jietang Zhao

**Affiliations:** State Key Laboratory for Conservation and Utilization of Subtropical Agro-bioresources/Key Laboratory of Biology and Genetic Improvement of Horticultural Crops (South China) of Ministry of Agriculture/Guangdong litchi engineering research center, College of Horticulture, South China Agricultural University, Guangzhou, China

**Keywords:** *Litchi chinensis*, *LcSWEETs*, Expression patterns, Seed development

## Abstract

**Background:**

SWEETs (Sugar Will Eventually be Exported transporters) function as sugar efflux transporters that perform diverse physiological functions, including phloem loading, nectar secretion, seed filling, and pathogen nutrition. The *SWEET* gene family has been identified and characterized in a number of plant species, but little is known about in *Litchi chinensis*, which is an important evergreen fruit crop.

**Results:**

In this study, 16 *LcSWEET* genes were identified and nominated according to its homologous genes in Arabidopsis and grapevine. Multiple sequence alignment showed that the 7 alpha-helical transmembrane domains (7-TMs) were basically conserved in LcSWEETs. The LcSWEETs were divided into four clades (Clade I to Clade IV) by phylogenetic tree analysis. A total of 8 predicted motifs were detected in the litchi *LcSWEET* genes. The 16 *LcSWEET* genes were unevenly distributed in 9 chromosomes and there was one pairs of segmental duplicated events by synteny analysis. The expression patterns of the 16 *LcSWEET* genes showed higher expression levels in reproductive organs. The temporal and spatial expression patterns of *LcSWEET2a* and *LcSWEET3b* indicated they play central roles during early seed development.

**Conclusions:**

The litchi genome contained 16 *SWEET* genes, and most of the genes were expressed in different tissues. Gene expression suggested that *LcSWEETs* played important roles in the growth and development of litchi fruits. Genes that regulate early seed development were preliminarily identified. This work provides a comprehensive understanding of the *SWEET* gene family in litchi, laying a strong foundation for further functional studies of *LcSWEET* genes and improvement of litchi fruits.

## Background

In higher plants, the major carbohydrate sucrose is transported to long-distances and regulation of its partitioning is necessary for plant development and stress responses [1, 2]. At present, three families of transporters have been characterized as key players in sugar translocation: the monosaccharide transporters (MSTs), the sucrose transporters (SUTs), and Sugar Will Eventually be Exported transporters (SWEETs) [3, 4]. The SWEET proteins function as sugar efflux transporters that transport hexose or sucrose across plasma membranes [5, 6]. They are characterized by the MtN3/saliva domain, which is also known as the PQ-loop repeat. MtN3/saliva domain comprises three alpha-helical transmembrane domains (3-TMs). Eukaryotic SWEETs typically consist of two tandemly repeated 3-TMs separated by a single TM, which constitutes a 7-TMs [7]. The distribution of carbohydrates is fundamental to crop yields. Therefore, it is important to explore the functions of SWEET proteins in plants.

SWEET proteins are considered to be involved in different sugar-efflux related processes, such as phloem loading, nectar secretion, supplying symbionts, and maternal efflux for filial tissue development [8]. Furthermore, SWEETs can be hijacked by pathogens for access to nutrition from hosts [9–12]. Indeed, SWEETs have been shown to affect various physiological processes, such as nectar secretion [13], pollen development [14, 15], modulating gibberellins response [16], senescence [17], cold tolerance [18, 19], seed and fruit development [20–22]. OsSWEET4 in rice and its ortholog ZmSWEET4c in maize have been reported to play important roles in grain filling [20].

Genome-wide identification and analysis of *SWEET* gene family have been reported in various plant species, such as *Arabidopsis thaliana* [5], *Oryza sativa* [23], *Vitis vinifera* [11], *Malus domestica* [24], *Citrus sinensis* [25], *Solanum lycopersicum* [26], *Glycine max* [27], *Brassica napus* [28], *S. tuberosum* [29], *Sorghum bicolor* [30], *Pyrus bretschneideri* [31], *Cucumis sativus* [32], *Musa acuminate* [33], *Hevea brasiliensis* [34], *Eriobotrya japonica* [35], *Camellia sinensis* [18], *B. oleracea* [19], *Ananas comosus* [21], *Triticum aestivum* [36], *B. rapa* [37], *Phalaenopsis equestris* and *Dendrobium officinale* [38]. In Arabidopsis, the 17 *SWEET* genes were classified into four clades according to the phylogenetic analysis: AtSWEET1–3 (Clade I), AtSWEET4–8 (Clade II), AtSWEET9–15 (Clade III), and AtSWEET16–17 (Clade IV) [5]. SWEETs in Clades I and II function mainly as glucose transporters [4, 5], Clade III are efficient sucrose transporters [6, 13], and Clade IV are located on tonoplast membrane and likely export fructose [4]. The *SWEET* genes in each clade may have similar functions, although they are versatile in different plants. At present, several *SWEET* genes have been identified in fruit trees [24, 25, 31, 35, 39], but their functions in fruit development are not clear.

Litchi (*Litchi chinensis* Sonn.) is an evergreen fruit crop, originated in China and commercially cultivated in the tropical and subtropical regions of the world. Litchi fruit has a succulent edible flesh (aril) surrounded by a red pericarp and a dark brown seed. Sugar composition in the aril of litchi fruit varies considerably among cultivars, based on different ratios of hexose/sucrose [40–42]. Moreover, the ratio of hexose/sucrose was significantly positively correlated with the weight of seeds [43]. There was no vascular tissue in the aril and the cotyledon, indicating an apoplasmic post-phloem sucrose transport from the funicle [44]. Extremely low activities of cell wall invertase (CWIN) were detected in the seed pedicel and seed coat of fruits with small seeds [45]. Silencing of *LcCWIN5* or *LcCWIN2* caused a reduction in the seed size [45]. It is common that CWIN and SWEETs co-expressed in the apoplasmic unloading region [46]. However, the functions of SWEETs in litchi seed development remain to be elucidated.

In this study, we comprehensively analyzed the gene structures, conserved motif compositions, chromosomal distribution of 16 *SWEET* genes in litchi. In addition, we studied the tissue-specific expression of *LcSWEETs* and their expression patterns during seed development between big-seeded and seed aborting cultivars. Moreover, the spatial expression of *LcSWEET2a*/*3b* in early seed development was analyzed. This study provides valuable information for screening important *SWEET* genes in litchi seed development.

## Results

### Identification and phylogenetic analysis of *SWEET* genes in litchi

**Fig. 1:**
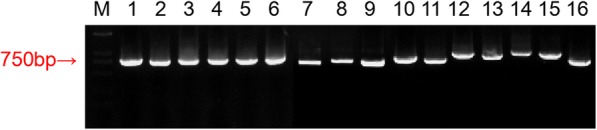
PCR amplification of full-length of the 16 *LcSWEET* genes in litchi. M: DNA 2000 marker; 1: *LcSWEET1*; 2: *LcSWEET2a*; 3: *LcSWEET2b*; 4: *LcSWEET3a*; 5: *LcSWEET3b*; 6: *LcSWEET4*; 7: *LcSWEET5*; 8: *LcSWEET6*; 9: *LcSWEET8*; 10: *LcSWEET9a*; 11: *LcSWEET9b*; 12: *LcSWEET10*; 13: *LcSWEET11*; 14: *LcSWEET12*; 15: *LcSWEET15*; 16: *LcSWEET17*

Arabidopsis *SWEET* genes were used as queries to find putative Litchi (*Litchi chinensis* Sonn.) *SWEET* genes. A total of 16 *SWEET* genes with two MtN3/saliva domains (PFAM motif PF03083) were obtained in litchi (Fig. 1, Additional file 1: Figure S1). Litchi *SWEET* genes, hereafter referred to as *LcSWEETs*, were named on the basis of their percentage of identity to the 17 Arabidopsis SWEET proteins. Gene characteristics, including the complete open reading frames (ORFs), GC content, molecular weight (MW), and isoelectric point (pI) were analyzed (Table 1). Among the 16 predicted LcSWEET proteins, LcSWEET9a was identified to be the smallest protein with 229 amino acid (aa), whereas the largest one was LcSWEET15 with 300 aa. The MW of the proteins ranged from 25.6 to 33.6 kDa, and the pI ranged from 7.66 (LcSWEET15) to 9.81 (LcSWEET9b) (Table 1).

**Table 1:** Characteristics of the 16 *LcSWEET* genes in litchi

Gene	GenBank number	Nucleotide sequence		Predicated polypeptide		
		ORF (bp)	GC content (%)	Length (aa)	Molecular weight (kDa)	pI
*LcSWEET1*	MN480570	750	45.8	249	27.4	9.70
*LcSWEET2a*	MN480571	708	39.9	235	26.0	8.56
*LcSWEET2b*	MN480572	729	38.0	242	26.9	9.64
*LcSWEET3a*	MN480573	750	42.0	249	28.3	9.47
*LcSWEET3b*	MN480574	755	42.6	251	28.2	9.06
*LcSWEET4*	MN480575	726	43.7	241	27.2	9.66
*LcSWEET5*	MN480576	714	43.1	237	26.4	9.18
*LcSWEET6*	MN480577	795	39.6	264	29.7	8.93
*LcSWEET8*	MN480578	720	45.3	239	26.4	9.01
*LcSWEET9a*	MN480579	690	40.8	229	25.6	9.29
*LcSWEET9b*	MN480580	720	41.1	239	26.9	9.81
*LcSWEET10*	MN480581	855	40.3	284	32.1	9.55
*LcSWEET11*	MN480582	813	49.7	270	30.2	9.21
*LcSWEET12*	MN480583	901	40.6	264	29.8	9.17
*LcSWEET15*	MN480584	903	40.9	300	33.6	7.66
*LcSWEET17*	MN480585	714	41.0	237	25.7	8.73

**Fig. 2:**
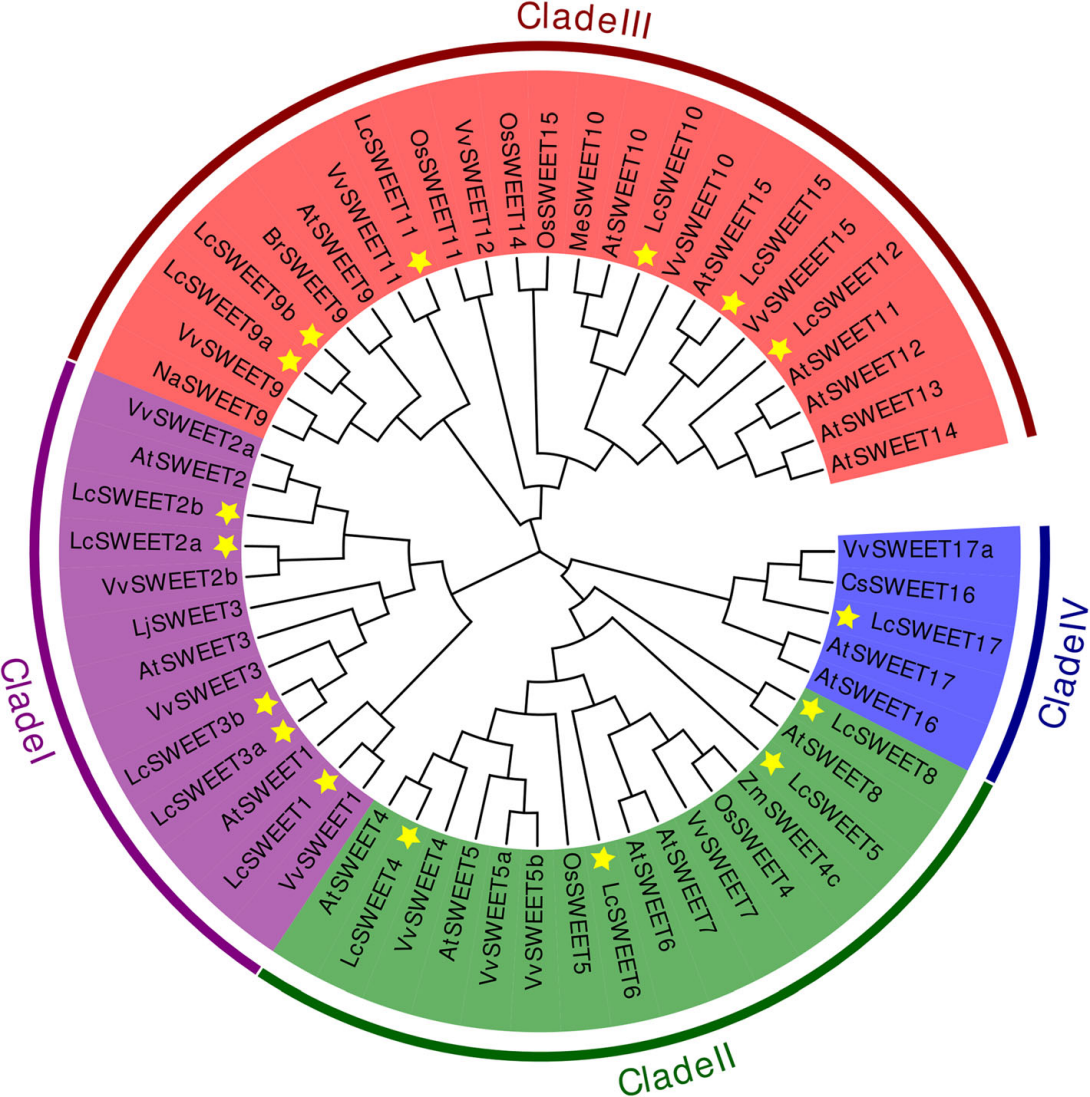
Neighbor-joining phylogenetic tree of SWEET proteins from litchi and other plant species. The roman numbers (I-IV) labeled with various colors indicate different clades. LcSWEETs are indicated by yellow star. The amino acid sequences and their accession numbers used to generate this phylogenetic tree are listed in Additional file 3: Table S1

Multiple sequence alignment of the 16 LcSWEET full-length protein sequences showed that the 7-TMs were basically conserved in specific positions (Additional file 2: Figure S2). In order to investigate the evolutionary relationships between LcSWEETs and SWEETs from other plant species, a neighbor-joining phylogenetic tree was constructed using MEGA 7 software. Results showed that the SWEET proteins could be clustered into four clades (Fig. 2). Clade I contained LcSWEET1/2a/2b/3a/3b; Clade II contained LcSWEET4/5/6/8; Clade III contained LcSWEET9a/9b/10/11/12/15; Clade IV contained LcSWEET17. LcSWEET3a/3b showed high sequence identity with LjSWEET3 from *Lotus japonicus* [47]. LcSWEET9a/9b had high sequence identity with AtSWEET9, BrSWEET9 and NaSWEET9 [13]. And LcSWEET10 shared high sequence identity with MeSWEET10 in cassava [48].

### Gene structure and conserved motif analysis of *LcSWEET*s

**Fig. 3:**
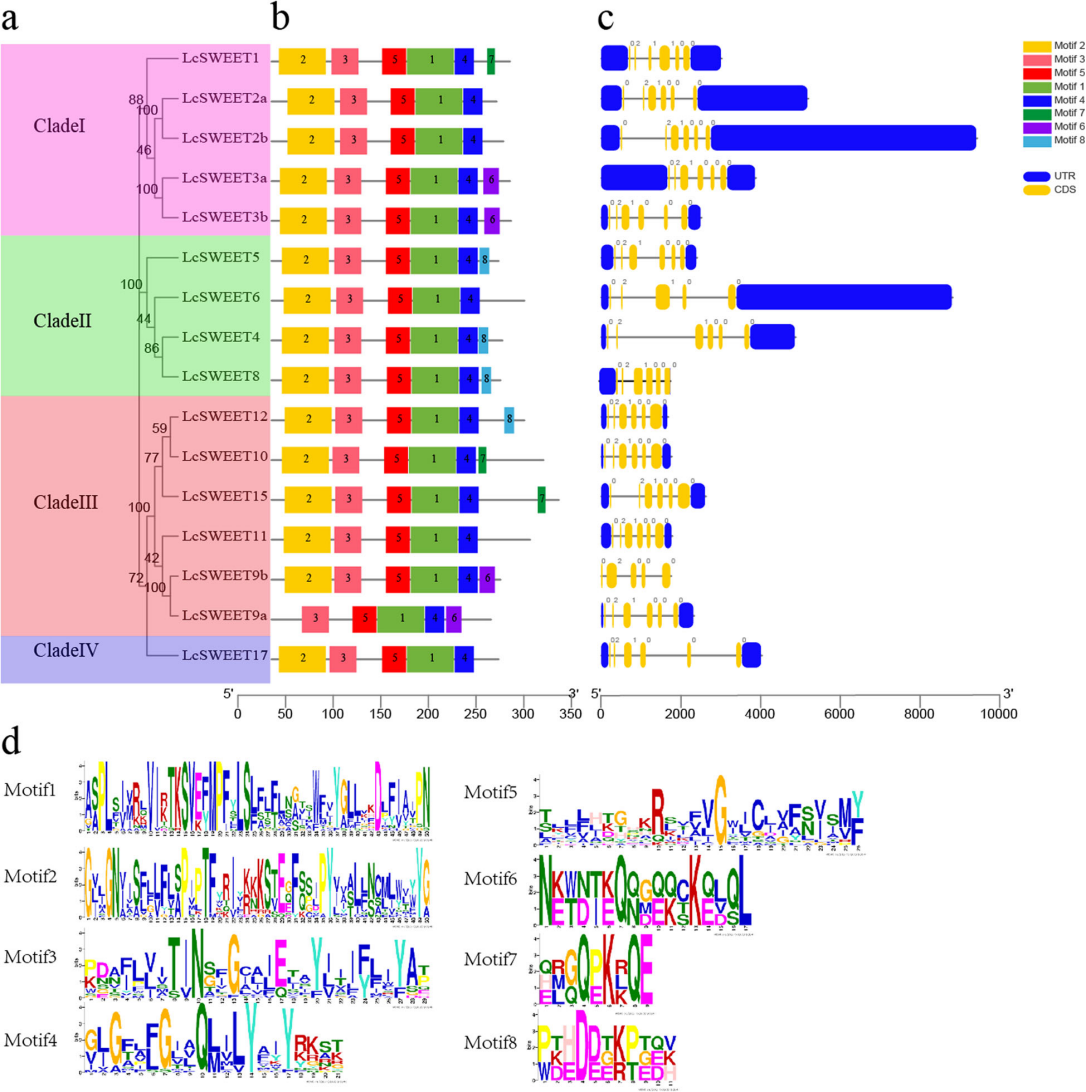
Phylogenetic relationship, gene structure and conserved motif analysis of *LcSWEET* genes. **a** The neighbor-joining phylogenetic tree was constructed with MEGA7 using amino acid sequences of LcSWEETs, and the bootstrap test replicate was set as 1000 times. **b** The motif composition of LcSWEET proteins. Five motifs were displayed in different colored rectangles. **c** Exon-intron structure of 16 *LcSWEET* genes. Yellow roundrectangles represent exons and black lines with same length represent introns. Blue roundrectangles indicate the UTR region. **d** The amino acid sequences of 8 motifs of LcSWEET proteins were showed

The exon-intron structures and conserved motifs were examined to gain more insight into the characteristic of *LcSWEET* genes. As shown in Fig. 3, most *LcSWEET* genes contained six exons, except *LcSWEET6* and *LcSWEET9b* containing five exons. Moreover, *LcSWEET* genes in the same Clade share similar gene structures in terms of exons location and introns length, although the introns length of *LcSWEET* members in Clade II and Clade IV were longer.

Furthermore, sequence motifs in 16 LcSWEETs were predicted using the MEME program. A total of 8 motifs were identified and named motif 1 to 8 (Fig. 3). Motif 1–5 were detected in all LcSWEET proteins except *LcSWEET9a* lacking Motif 2. Motif 6 was detected in *LcSWEET3a*, *LcSWEET3b*, *LcSWEET9a*, and *LcSWEET9b*. Motif 7 was detected in *LcSWEET1*, *LcSWEET10*, and *LcSWEET15*. Motif 8 was only detected in *LcSWEET4, LcSWEET8, LcSWEET5* and *LcSWEET12*. Overall, the conserved motif in the N-terminus of all LcSWEET proteins was similar.

### Chromosomal localization and synteny analysis of *LcSWEET*s

**Fig. 4:**
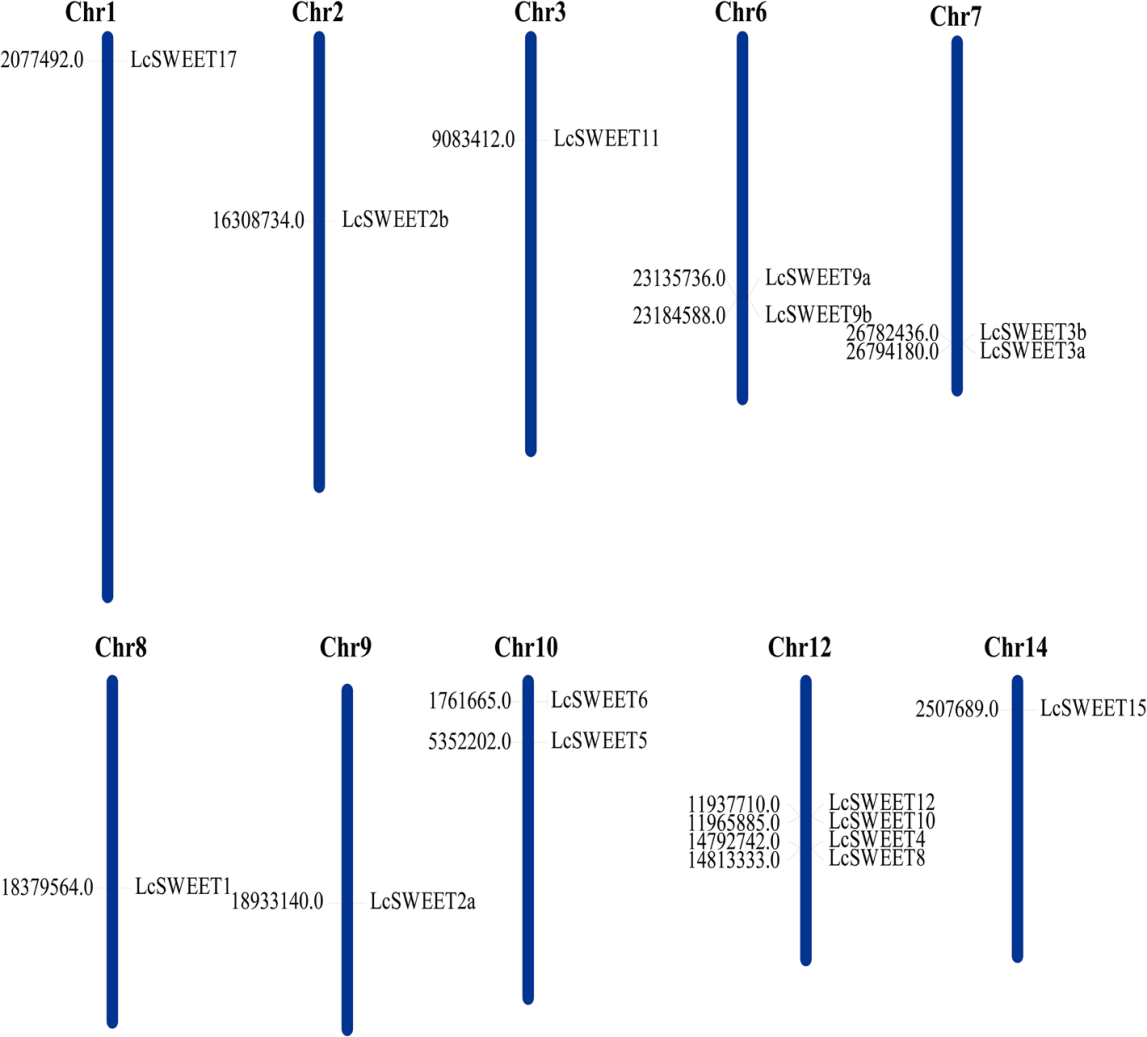
Schematic representations of the chromosomal location of the *LcSWEET* genes. The chromosome number is indicated on the top of each chromosome

**Fig. 5:**
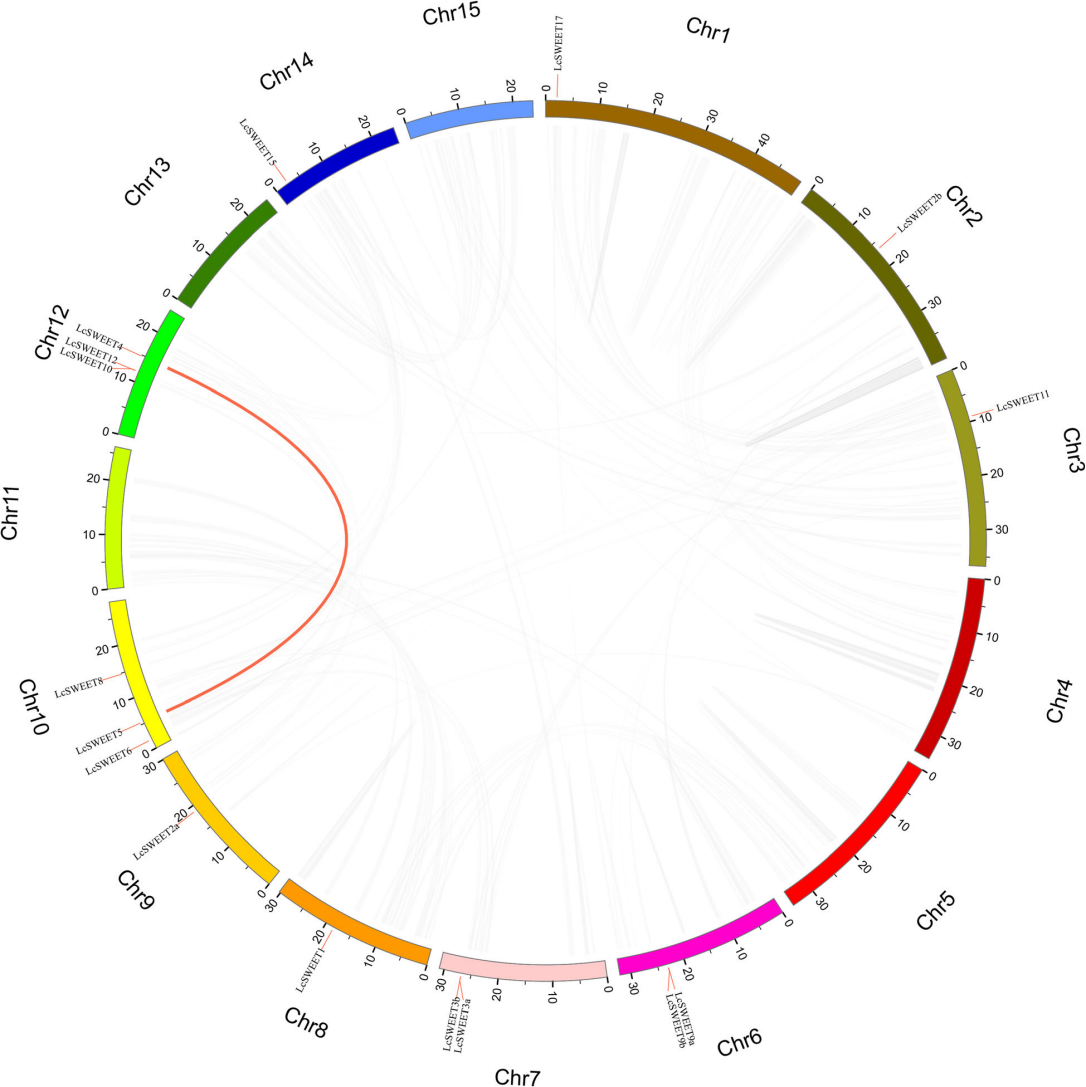
The synteny analysis of *LcSWEET* genes. Fifteen chromosomes were drawn in different colors. The chromosome location of *LcSWEET* genes were shown by short red lines on the circle. Gray lines indicate all synteny blocks in the litchi genome, and the red lines indicate the duplication of *LcSWEET* gene pairs

According to the gene loci information, the 16 *LcSWEET* genes were unevenly distributed in 9 chromosomes and the detailed chromosomal locations were shown in the Fig. 4. Most of *LcSWEET* members were dispersed to different chromosomes. However, there were 4 *LcSWEET* genes in Chromosome 12. After the synteny analysis of *LcSWEETs*, there was one pairs of segmental duplicated events (Fig. 5). *LcSWEET4* and *LcSWEET5* may be generated by fragment duplication. *LcSWEET3a/3b, LcSWEET9a/9b and LcSEET10/12* were clustered into 3 tandem duplication events with BLASTP and MCScanX methods. Based on the above results, some *LcSWEET* genes were probability generated by gene segmental or tandem duplication.

### Tissue-specific expression of *LcSWEET* genes

**Fig. 6:**
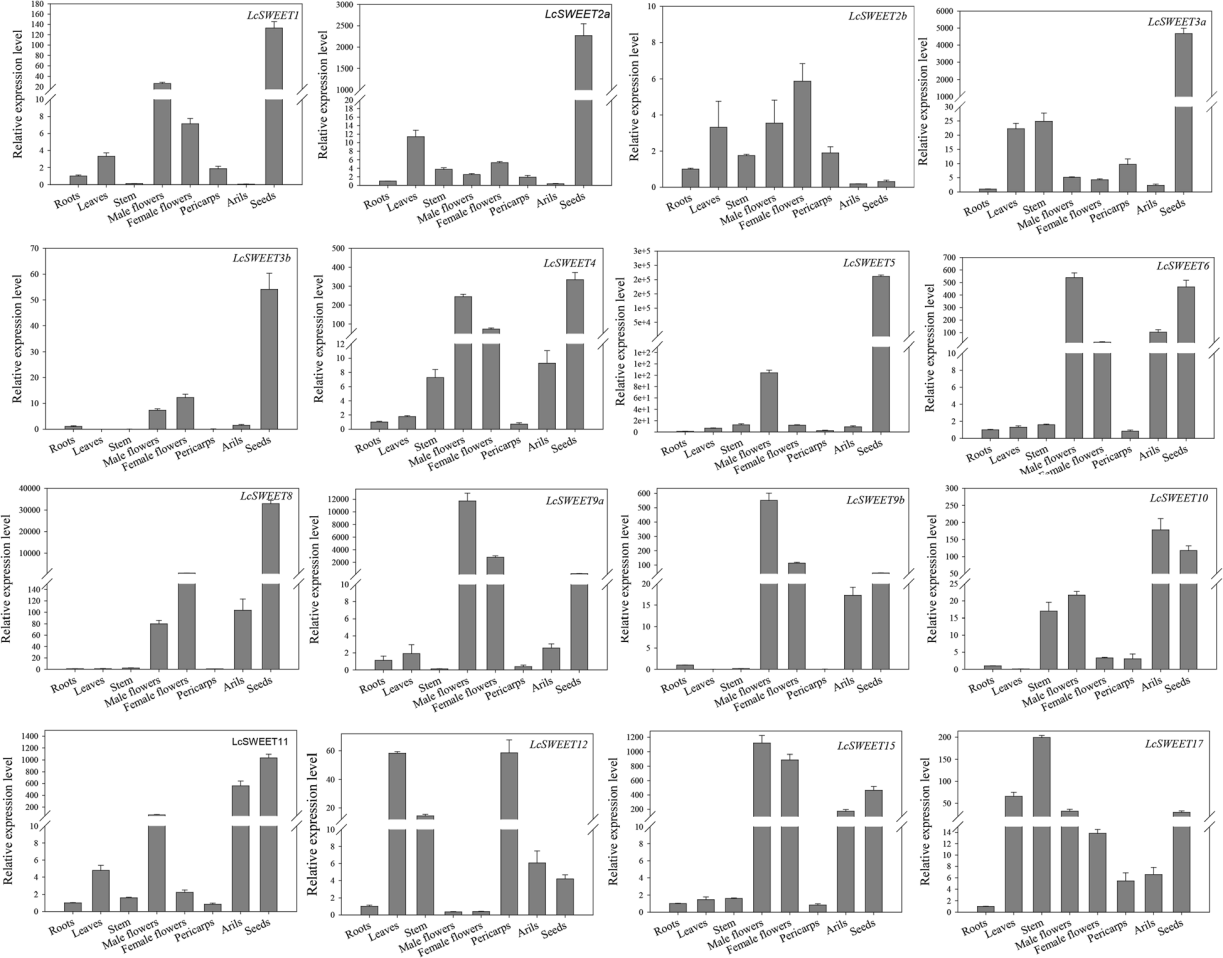
Quantitative real-time PCR analysis of the expression levels of 16 *LcSWEET* genes in in different litchi tissues. *LcActin* (HQ615689) was used as an internal control. The vertical bars indicate the standard error of three replicates

To obtain insights into the physiological functions of the *LcSWEET* genes, the expression patterns of each *LcSWEET* gene in different tissues (leaves, stems, roots, male flowers, female flowers, pericarps, arils, and seeds) were measured by RT-qPCR assay. The results showed that the expression pattern differed among the 16 *LcSWEET* genes (Fig. 6). Most of the *LcSWEET* genes showed higher expression levels in reproductive organs. However, *LcSWEET2b* was almost not detected in the arils and seeds, and *LcSWEET12*/*17* had high expression levels in leaves and stems. The expression levels of *LcSWEET1*/*2a*/*3a*/*3b*/*4*/*5*/*8*/*11* in the seeds were the highest among different tissues. Moreover, *LcSWEET2a*/*3a*/*3b*/*5* were mainly expressed in seeds. *LcSWEET6*/*9a*/*9b*/*15* showed higher expression levels in flowers, arils and seeds. Interestingly, the expression levels of these genes in male flowers were higher than those in female flowers. *LcSWEET10* had the highest expression level in arils and *LcSWEET17* showed the highest expression level in stems. *LcSWEET12* was expressed at high levels in leaves and pericarps.

### Expression patterns of *LcSWEET* genes during seed development

**Fig. 7:**
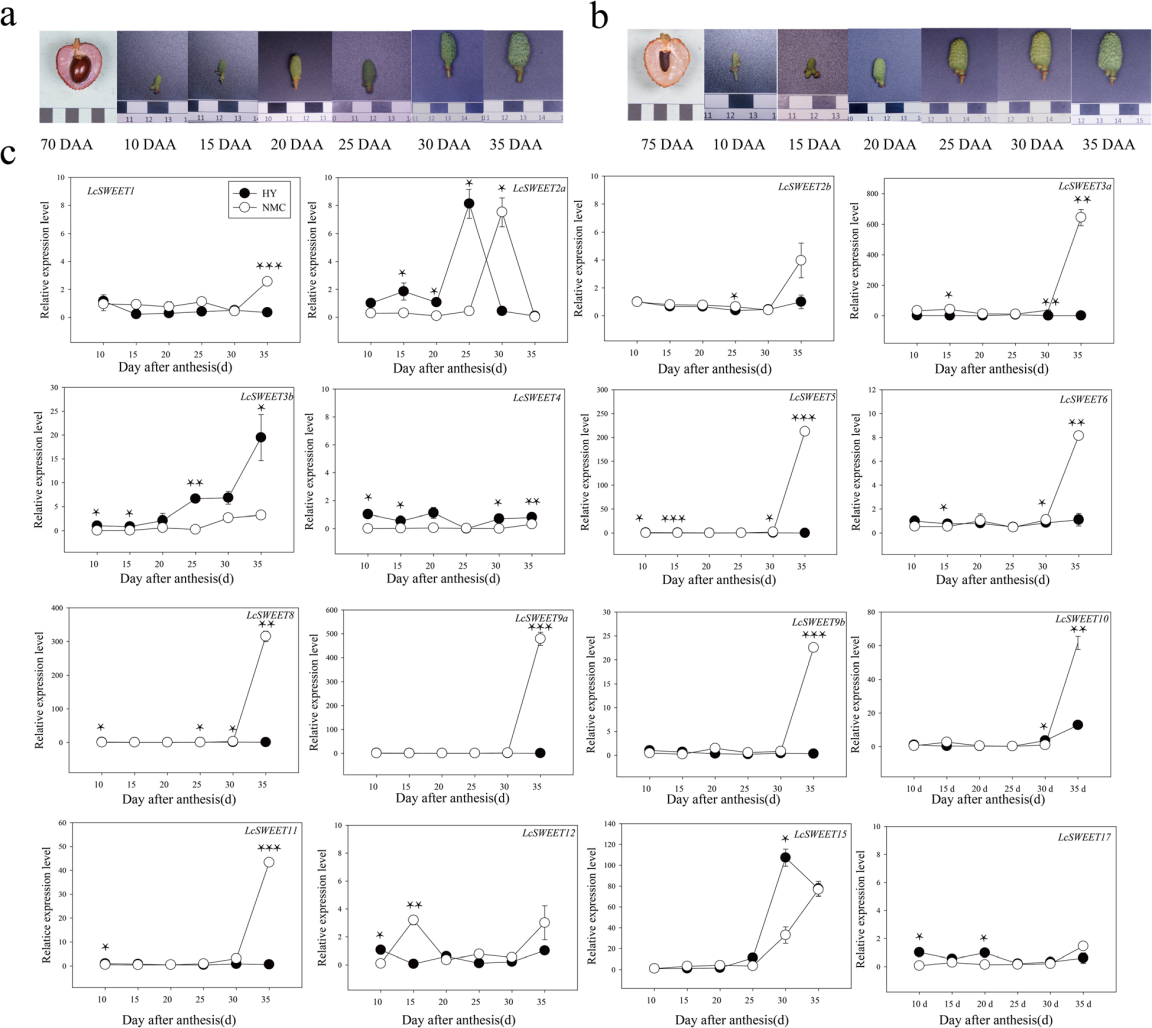
Changes in the fruit development of HY (**a**), NMC (**b**), and the expression of *LcSWEET* genes during seed development of HY and NMC as determined by quantitative real-time PCR (**c**). *LcActin* (HQ615689) was used to normalize gene expression. Asterisks denote a significant difference during seed development between HY and NMC samples (*p < 0.05, ** *p* < 0.01, *** *p* < 0.001). The error bars represent the standard error of three replicates

Fruit with a small seed is an economically desirable trait of litchi fruits, and seed size has been found to be associated with the expression levels of *LcCWINs*, which might affect seed development through sugar import and/or sugar signaling [45]. According to our previous study [45], 28 days after anthesis (DAA) represents a transition point between the cell division stage and the filling stage during litchi seed development. As the seed size has been determined at the cell division stage. We systematically studied the expression of *LcSWEET* genes at early stages of seed development. The expression patterns of *LcSWEET* genes at six developmental stages were different between big-seeded cultivar ‘Heiye’ (HY) and seed-aborting cultivar ‘Nuomici’ (NMC) (Fig. 7). The expression of *LcSWEET1*/*4*/*12*/*17* remained low in both big-seeded and seed-aborting cultivars through the six developmental stages. A significant increasing was observed in the expression of *LcSWEET2b*/*3a*/*5*/*6*/*8*/*9a*/*9b*/*11* at 35 DAA in NMC, which was not observed in HY. The expression of *LcSWEET10*/*15* increased at 30 DAA in both HY and NMC. Two *LcSWEET* genes, *LcSWEET2a* and *LcSWEET3b*, were found to be correlated with early seed development. The expression of *LcSWEET2a* peaked at 25 DAA in HY, which was earlier than that in NMC. The expression of *LcSWEET3b* was higher in HY than that in NMC. With the seed development, a noticeable increased expression of *LcSWEET3b* was observed since 20 DAA in HY, however, the levels of this gene in NMC were just slightly elevated.

### Spatial patterning of *LcSWEET2a* and *LcSWEET3b* in early seed development

**Fig. 8:**
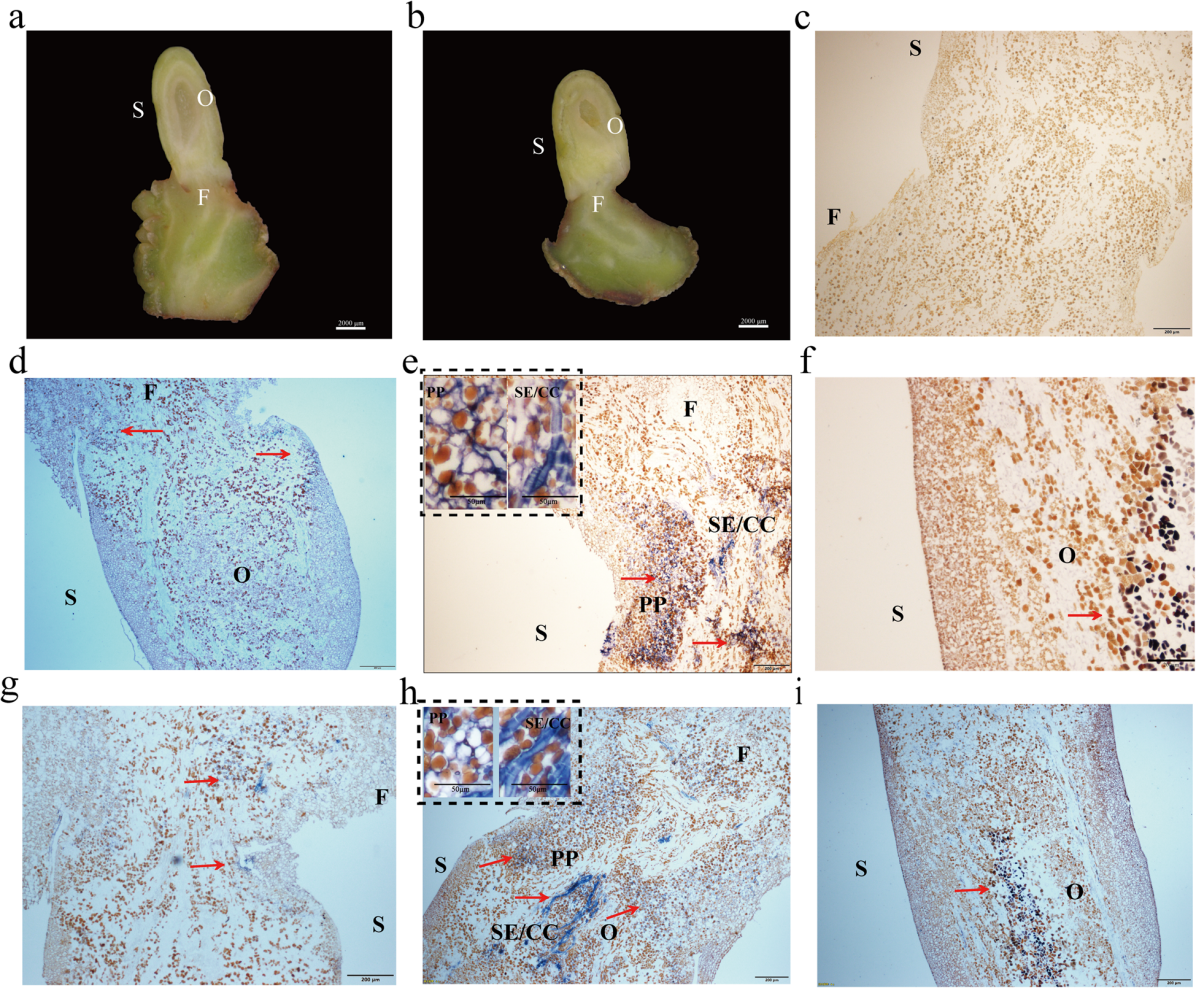
Spatial distribution of *LcSWEET2a* and *LcSWEET3b* mRNA in 15 DAA funicle and ovule of HY and NMC. Longitudinal sections of 15 DAA seed of HY (**a**) and NMC (**b**) were viewed under bright field. Longitudinal sections of 15 DAA funicle and ovule with sense RNA probes (**c**). Longitudinal sections of 15 DAA funicle and ovule of NMC (**d**) and HY (**e, f**) hybridized with antisense RNA probes for *LcSWEET2a*. Longitudinal sections of 15 DAA funicle and ovule of NMC (**g**) and HY (**h**, **i**) hybridized with antisense RNA probes for *LcSWEET3b*. Magnified views of the SE/CC and PP were inside the black virtual frame on the left top corner of (**e** and **h**). Red arrows indicate the *LcSWEET2a* and *LcSWEET3b* signals. S, seed; O, ovule; F, funicle. SE/CC, Sieve element-companion cell. PP, parenchyma cells. Bars = 2000 μm (**a, b**), 200 μm (**c-i**), 50 μm (magnified views of the SE/CC and PP)

According to Lü et al. [49], the seed at 15 DAA was the critical stage of early embryonic development in litchi. Our previous study indicated funicle was the site of sucrose unloading [44]. Therefore, we conducted in situ hybridization experiments on the funicle and seed at 15 DAA to further explore the roles of *LcSWEET2a* and *LcSWEET3b* in early seed development. The results indicated that the spatial distribution of *LcSWEET2a* and *LcSWEET3b* transcripts appeared more restrictive to specific areas (Fig. 8). In HY, abundant *LcSWEET2a* and *LcSWEET3b* were expressed in the funicle and ovule in comparison with the sense control. However, only little *LcSWEET2a* and *LcSWEET3b* transcripts were detected in the funicle and no transcript signals were found in the ovule of NMC. Thus, the different spatial patterning of *LcSWEET3b* and *LcSWEET2a* between NMC and HY indicated their involvement in the early seed development in litchi.

## Discussion

### *SWEET* gene family in litchi

Plant SWEET proteins play critical roles in growth, development, and stress responses by regulation of sugar transport and distribution [4, 8]. Recently, *SWEET* gene families have been analyzed from over 20 plant species. Increasing evidence indicates SWEETs play important roles in fruit development and ripening [21, 22]. However, the *SWEET* gene family has not been studied in litchi. In this study, we identified and characterized *SWEET* gene family in litchi through genome-wide analyses, and further investigated their expression patterns during early seed development.

In higher plants, the number of reported *SWEET* genes varies from 7 to 108, with 17 in Arabidopsis [5], 17 in grapevine [11], 21 in rice [23], and 108 in wheat [50]. In the present study, 16 *LcSWEET* genes were isolated from litchi. The varied number of *SWEET* genes in different plant species may be evolved from tandem or segmental duplication [27, 50, 51]. Indeed, one segmental duplicated event (*LcSWEET4* and *LcSWEET5*) and 3 tandem duplication events (*LcSWEET3a*/*3b*, *LcSWEET9a*/*9b* and *LcSEET10*/*12*) were identified in the present study (Figs. 4, 5). The length of LcSWEET proteins ranged from 229 aa to 300 aa, which was similar to that has been reported in other plants, such as 233–308 aa in tomato [26], 171–333 aa in banana [33], and 215–340 aa in apple [39].

Genomic structural analysis showed that majority of *LcSWEET* genes consisted of six exons except *LcSWEET6* and *LcSWEET9b* (Fig. 3). Similar results were earlier reported in Chinese white pear [31], banana [33], and apple [39]. Chen et al. [5] carried out phylogenetic analysis of Arabidopsis *SWEET* genes and divided them into four clades. Our results support *LcSWEET* genes could be classified into four clades. Clade I, II, III, and IV contained 4, 4, 6, and 1 *LcSWEET* genes, respectively. In the previous studies, it has been shown that phylogenetic analysis also supports the results of conserved motif analysis [33, 50]. Our results indicated that gene members in each clade harbored some unique conserved motif (Fig. 3), indicating they might have different functions in litchi.

### Expression and function diversity of *SWEET* genes in litchi

Plant *SWEET* genes are found to be differentially expressed in tissues and are involved in different sugar transport [52]. Differential expression analysis of *SWEET* genes in litchi has implement us to find out specialized functions of each SWEET protein in sugar transport. In this study, most of the *LcSWEET* genes were highly expressed in flowers and seeds (Fig. 6), which are strong carbon sinks. However, the expression patterns of the pairs of duplicated genes differed in litchi (Figs. 6, 7). For example, *LcSWEET3a*/*3b* had different expression levels during early seed development, whereas *LcSWEET9a*/*9b* had similar expression patterns in flowers and seeds. Our results are consistent with results reported for apple [39] and wheat [50]. These results suggest that the *SWEET* genes in litchi have undergone duplication and some duplicated *SWEET* genes have diverged in functions.

*LcSWEET2a* and *LcSWEET3b* were mainly expressed in seeds, and may play important roles in sugar partitioning during seed development. The physiological roles of *LcSWEET* genes in seed development were further discussed below in detail. *SWEET9* has been identified as nectary-specific in *Arabidopsis*, *Brassica*, and *Nicotiana*, and function as a sucrose transporter for nectar production [13]. In this study, *LcSWEET9a*/*9b* showed high expression levels in flower. And LcSWEET9a/9b was shown to share high sequence identity with AtSWEET9, BrSWEET9 and NaSWEET9 (Fig. 2). These results suggested that LcSWEET9a/9b appeared to be responsible for nectar secretion in litchi. The transcript levels of *LcSWEET6*/*15* were higher in the flowers, arils and seeds than in other tissues, indicating the function of these genes in specialized organs. Similar studies found that the expression levels of Arabidopsis *AtSWEET15* and tea *CsSWEET15* were high in flowers and seeds [18, 53]. *LcSWEET10* was expressed at high levels in aril. A previous work revealed that grapevine *VvSWEET10* was strongly expressed at the onset of ripening and can improve grape sugar content [22]. Overexpression of *VvSWEET10* in grapevine callus and tomato increased the sugar levels significantly [22]. The potential role of *LcSWEET10* in litchi fruit sugar accumulation is worthy to make further studies.

### LcSWEET2a/3b are involved in early seed development in litchi

SWEET transporters are known to transfer assimilate from maternal tissues to developing seeds [4], supplying nutritional and signaling sugars for seed development. The roles of SWEET transporters in seeds were demonstrated during seed filling stage. In Arabidopsis, *AtSWEET11*, *AtSWEET12* and *AtSWEET15* were expressed in both seed coat and endosperm, and the *AtSWEET11*, *AtSWEET12* and *AtSWEET15* triple mutant showed delayed embryo development and seed shrinkage [53]. Notably, the rice *OsSWEET11* and *OsSWEET15* transporters both contribute to seed filling with seemingly redundant roles [54]. Overall, SWEET transporters in Clade III responsible for importing sucrose play central roles in seed filling. However, the function of SWEET transporters in early seed development was unclear. In our previous study, the seed development in litchi could be clearly divided into the cell division stage and the filling stage around 28 DAA [45]. Here we performed the temporal and spatial expression patterns of *LcSWEET2a* and *LcSWEET3b* during early seed development in litchi. Clearly, the big-seeded cultivar HY showed higher expression levels of *LcSWEET2a* and *LcSWEET3b* than those in seed-aborting cultivar NMC. Moreover, the expression sites of *LcSWEET2a* and *LcSWEET3b* in HY concentrated in the funicle and ovule, which are the sites of apoplasmic phloem unloading of sucrose [44]. Therefore, we speculate that *LcSWEET2a* and *LcSWEET3b* may cooperate with CWIN in hexose import into liquid endosperm. Indeed, we previously found significant lower levels of CWIN protein and activity associated with seed abortion in NMC [45]. During early seed development, CWIN hydrolyze incoming sucrose into hexoses, and the adjacent expression of hexose transporters will be predicted. SWEETs in Clade I and II as hexose transporters may be involved in importation of CWIN-derived hexoses into endosperm. In maize, *ZmSWEET4c* was expressed in the basal endosperm transfer layer (BETL) and necessary for BETL differentiation [20]. Because *OsSWEET4* was predominantly expressed at early stages of seed development, it appears to have similar roles as *ZmSWEET4c* [20]. The hexoses produced from CWIN and SWEET transporters are thought to stimulate mitotic activity to increase cell number [55]. Taken together, our finding strongly indicates that *LcSWEET2a* and *LcSWEET3b* function in determining the seed abortion or seed size by direct translocation of carbohydrates.

## Conclusions

In summary, our work firstly analyzed the *SWEET* gene family in litchi. Sixteen *LcSWEET* genes were isolated and comprehensively characterized, including gene structures, conserved motifs, chromosomal distribution, gene duplication, evolutionary relationships, spatial and temporal expression patterns. The phylogenetic tree divided the *LcSWEET* gene into four clades, each of which had similar gene structures and motif compositions. Tissue-specific expression patterns suggested the function diversity of these *LcSWEET* genes. The temporal and spatial expression patterns of *LcSWEET2a* and *LcSWEET3b* suggested their central roles during early seed development. Our research lays a foundation for the elaboration of the functions of the *LcSWEET* genes in the growth and development of litchi fruits.

## Methods

### Plant materials

Litchi (*Litchi chinensis* Sonn.) cultivars ‘Heiye’ (HY), and ‘Nuomici’ (NMC) were grown in the orchard of South China Agricultural University, Guangzhou, China. Trees used for samples collection were under the same integrated orchard management practices. Leaves, stems, roots, male flowers, female flowers, pericarps, arils, and seeds of FZX were collected for gene cloning and tissue-specific gene expression. For gene expression in early seed development, 6 stages of seeds of HY and NMC from 10 DAA until 35 DAA at 5 days interval were sampled. For in situ hybridization experiments, HY and NMC seeds at 15 DAA were collected. Ten uniformly sized fruits were sampled at every stage (one replicate). Three replicates were collected. All samples were immediately frozen in liquid nitrogen and kept at − 80 °C before use.

### Identification and molecular cloning of *SWEET* gene family in litchi

Litchi *SWEET* gene family were identified by protein Blast of the 17 Arabidopsis SWEET proteins against Litchi genome database (unpublished). The conserved domains of LcSWEET proteins were identified by using NCBI-CCD (https://www.ncbi.nlm.nih.gov/cdd).

Total RNA was extracted using the Quick RNA isolation Kit (Huayueyang, Beijing, China) and the first-strand cDNA was synthesized using Scientific RevertAid First Strand cDNA Synthesis Kit (Thermo Fisher Scientific, USA). The coding sequences of *LcSWEET* genes were amplified from cDNA using gene-specific primers (Additional file 4: Table S2). Phanta Max Super-Fidelity DNA Polymerase (Vazyme, Nanjing, China) was used for PCR amplification. The purified PCR products were ligated into the pEASY-Blunt Cloning Vector (TransGen, Beijing, China) for sequencing.

### Sequence analysis

Multiple sequence alignments of the amino acid sequences were generated using DNAMAN software and TMHMM server version 2.0 (http://www.cbs.dtu.dk/services/TMHMM/). The number of amino acids, molecular weights, and theoretical pI were analyzed on the ExPASy website (http://web.expasy.org/potparam/).

### Phylogenetic analysis

The full-length amino acid sequences of SWEETs for phylogenetic tree were downloaded from TAIR (https://www.arabidopsis.org/) and NCBI database (https://www.ncbi.nlm.nih.gov), respectively. A neighbor-joining phylogenetic tree were constructed using MEGA7 software with bootstrap test of 1000 times [56]. Then, the phylogenetic tree was annotated with EVOLVIEW (http://120.202.110.254:8280/evolview) [57].

### Gene structure analysis and identification of conserved motifs

The exon-intron structures were analyzed by TBtools software (http://www.tbtools.com/) and the MEME (http://meme-suite.org/) website was used for conserved protein motif analysis.

### Chromosomal distribution and gene synteny analysis

The location information of *LcSWEET* genes were obtained from the Litchi genome database (unpublished). The gene location map was constructed using MapChart [58]. The synteny analysis was constructed using the MCScanX and CRCOS [59].

### RT-qPCR analysis

RT-qPCR was conducted to determine the expression profile for each member of the *LcSWEET* genes using various tissues and developmental stages seeds. RT-qPCR expression analysis was carried out using our established protocol [60]. All reactions were performed in triplicate with three biological replicates. Gene primer sequences for RT-qPCR were listed in Additional file 5: Table S3.

### Statistical analysis

All the reported values were expressed as mean ± standard error (SE). Significant differences were determined with a T-TEST using IBM SPSS Statistics software 19.0 (SPSS Inc., USA) for Windows.

### In situ hybridizations

In situ hybridization was carried out using the protocol of Jackson [61] with some modifications. The funicle and seed of 15 DAA of HY and NMC were fixed in 10% Neutral Formalin Fix Solution (NBF). The samples were rinsed twice each in 1× PBS on ice for 15 min. The samples were dehydrated in a graded ethanol series. To transparent, we replaced the ethanol gradually with xylene. The samples were infiltrated with Paraplast Plus at Paraplat:xylene volume ratio of 2:1, 1:1, 2:1 for 1 h in a 60 °C oven at each step. Lastly, the samples were embedded in 100% Paraplast at 60 °C overnight and stored at 4 °C.

Probes for in situ hybridization were amplified using cDNA from funicle and seed at 15 DAA as template. Sense and antisense RNA probes were synthesized using a DIG RNA labeling mix (Roche Diagnostics, USA). To generate gene-specific probes of *LcSWEET2a*, a 140 bp fragment, was amplified using *LcSWEET2a*F, 5′-GAAAGGCAGATTTTTGTTGG-3′, and *LcSWEET2a*R, 5′ -AAGAAGGTTGAAAGCGAGAG-3′. To generate gene-specific probes of *LcSWEET3b*, a 172 bp fragment, was amplified using the primer set of *LcSWEET3b*F, 5′-ATGACCTATGGACTACTGG-3′, and *LcSWEET3b*R, 5′ -GCTGTTTGGACTTCTCCAT-3′. Fluorescent microscope (ZEISS) was used to observe the samples.

## Supplementary information

**Additional file 1: Figure S1.** The MtN3/saliva domain of LcSWEETs based on the entire protein sequences using NCBI CCD (https://www.ncbi.nlm.nih.gov/cdd).

**Additional file 2: Figure S2.** Alignment of 16 LcSWEET protein sequences. Highly conserved residues are indicated in color and the seven transmenbrane domains are indicated with TM.

**Additional file 3: Table S1.** The accession number and amino acid sequences used to generate phylogenetic tree.

**Additional file 4: Table S2.** Primers used for SWEET genes isolation.

**Additional file 5: Table S3.** Primers used for RT-qPCR.

## Data Availability

The data that support the results are included within the article and its additional file.

## Abbreviations

aa: Amino acid
CWIN: Cell wall invertase
DAA: Days after anthesis
MSTs: Monosaccharide transporters
MW: Molecular weight
NBF: Neutral Formalin Fix Solution
ORFs: Open reading frames
pI: Isoelectric point
RT-qPCR: Reverse transcription quantitative real-time polymerase chain reaction
SUTs: Sucrose transporters
SWEETs: Sugar Will Eventually be Exported transporters
TMs: Alpha-helical transmembrane domains
