# Supplementary figures and images for: Genome-wide identification and expression analysis of SWEET gene family in Litchi chinensis reveal the involvement of LcSWEET2a/3b in early seed development
Source: BMC Plant Biol. 2019 Nov 14;19:499. doi: 10.1186/s12870-019-2120-4 (PMC6857300; doi:10.1186/s12870-019-2120-4)

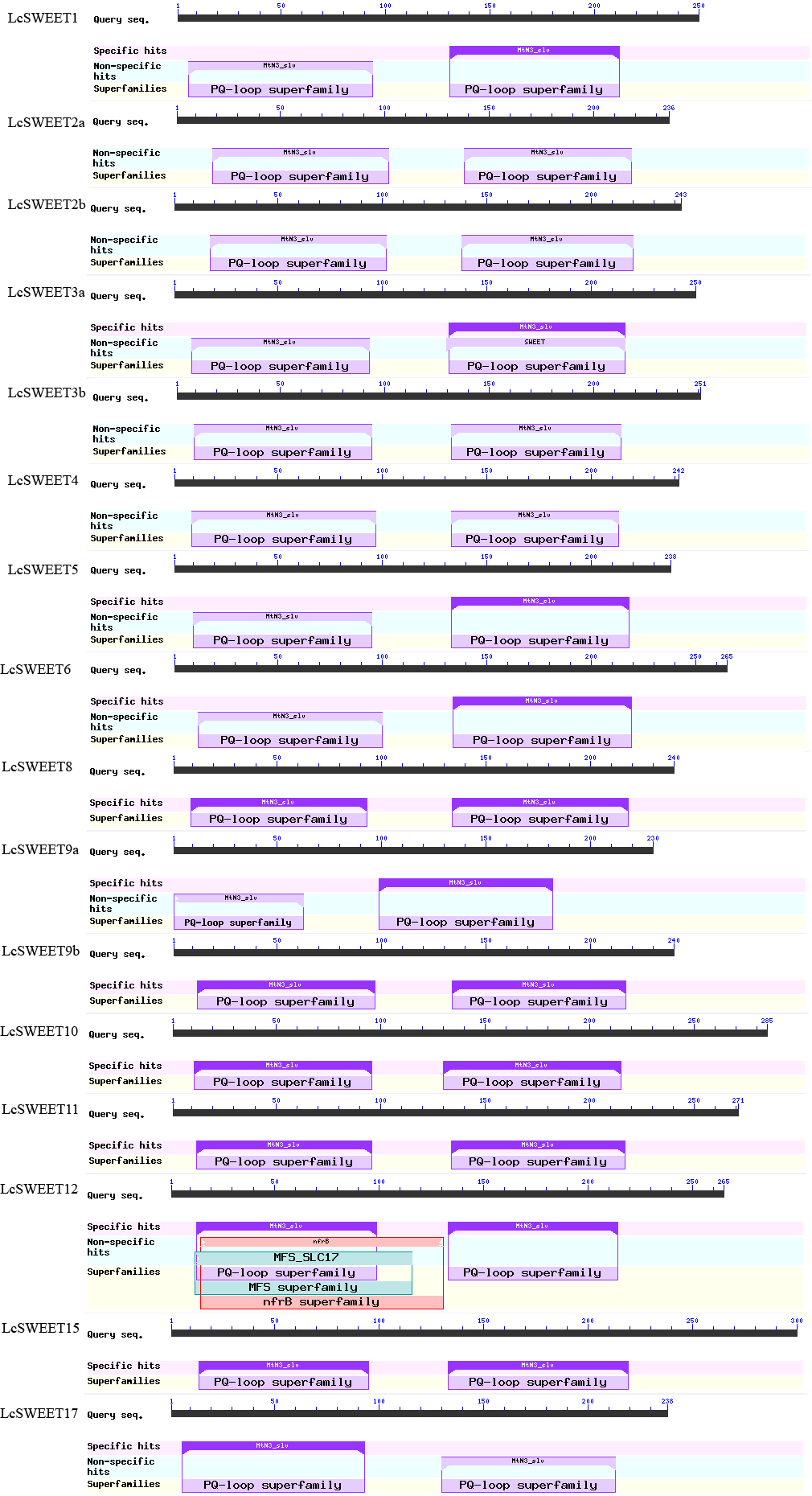

Supplement: Supplementary file 1 — Additional file 1: Figure S1. The MtN3/saliva domain of LcSWEETs based on the entire protein sequences using NCBI CCD (https://www.ncbi.nlm.nih.gov/cdd). [file 12870_2019_2120_MOESM1_ESM.tif]

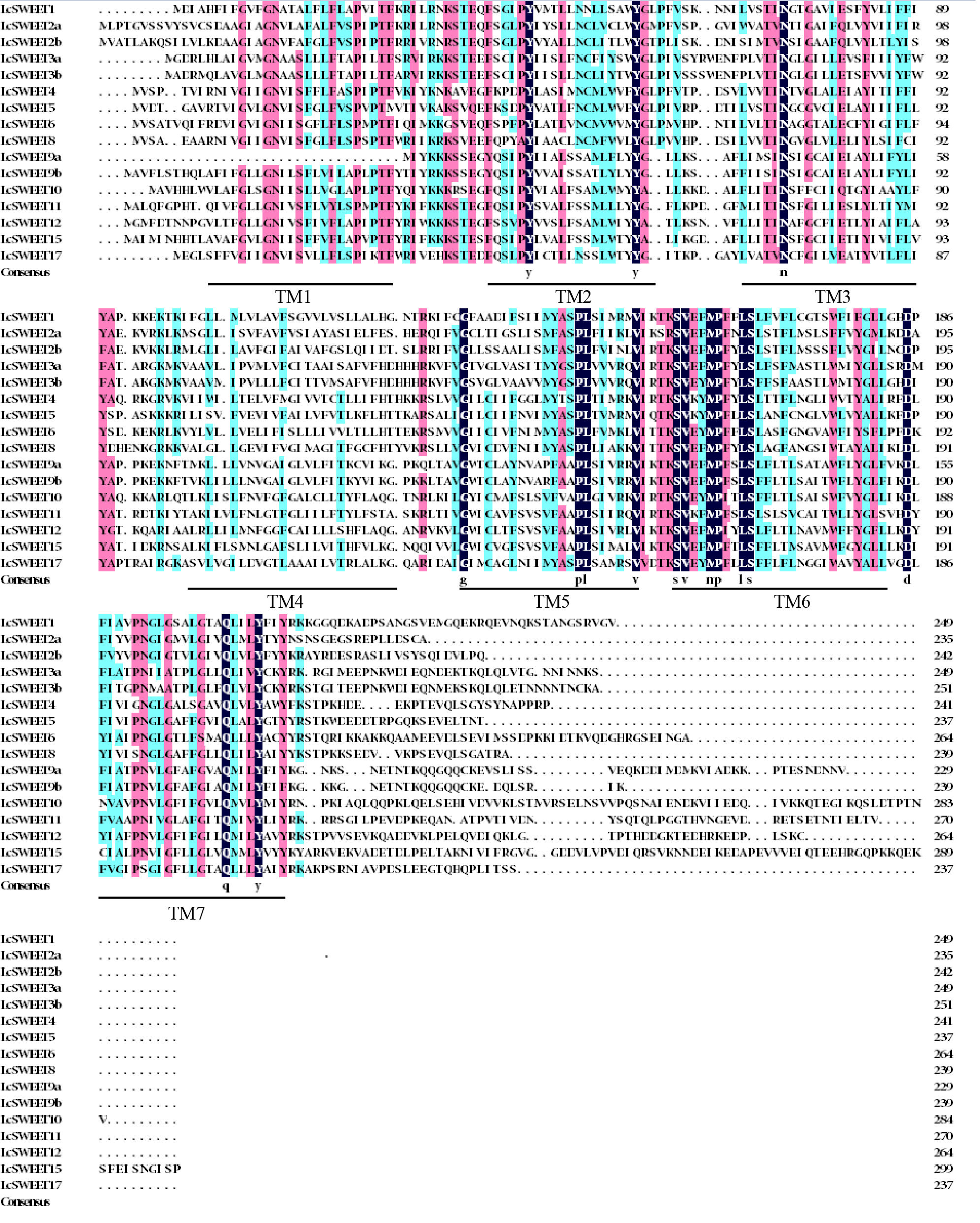

Supplement: Supplementary file 2 — Additional file 2: Figure S2. Alignment of 16 LcSWEET protein sequences. Highly conserved residues are indicated in color and the seven transmenbrane domains are indicated with TM. [file 12870_2019_2120_MOESM2_ESM.tif]
